# Bilateral Meralgia Paresthetica With Concurrent Right Piriformis Syndrome Following a Febrile Illness: A Case Report

**DOI:** 10.7759/cureus.109719

**Published:** 2026-05-27

**Authors:** Kyaw Zin Aung, Thinn Thiri Soe, Cherry Myint, Lin Swe Htet, Phyo Thant, Naw Eh Law Saw, Su Su Htun, Ei Ei Cho

**Affiliations:** 1 Internal Medicine, Kulhudhuffushi Regional Hospital, Kulhudhuffushi, MDV; 2 Emergency Department, Kulhudhuffushi Regional Hospital, Kulhudhuffushi, MDV; 3 Internal Medicine, Royal College of Physicians of the United Kingdom, Edinburgh, GBR; 4 Internal Medicine, Kanditheemu Health Centre, Kanditheemu, MDV; 5 Orthopaedic Surgery, University of Medicine 1 Yangon, Yangon, MMR

**Keywords:** lateral femoral cutaneous nerve, meralgia paresthetica, nerve conduction study, piriformis syndrome, post-infectious neuropathy

## Abstract

Meralgia paresthetica (MP) is an entrapment neuropathy of the lateral femoral cutaneous nerve (LFCN) that is typically caused by mechanical compression. Piriformis syndrome (PS) is an uncommon neuromuscular disorder resulting from sciatic nerve compression or irritation by the piriformis muscle. The concurrent occurrence of MP and PS is rare and may pose significant diagnostic challenges because of overlapping sensory and radicular-like symptoms. We report the case of a 30-year-old previously healthy man who developed bilateral burning pain over the anterolateral thighs and right gluteal discomfort following a self-limiting febrile illness. Neurological examination demonstrated sensory dysesthesia in the LFCN distribution and a positive flexion-adduction-internal rotation (FAIR) test on the right side. Nerve conduction studies demonstrated bilateral LFCN conduction abnormalities consistent with MP, while pelvic magnetic resonance imaging demonstrated edema and hypertrophy of the right piriformis muscle. The patient demonstrated significant clinical improvement following treatment with pregabalin, nonsteroidal anti-inflammatory drugs, and physiotherapy. This case highlights the importance of considering a possible post-infectious inflammatory association and coexisting entrapment neuropathies in patients presenting with atypical lower-limb sensory symptoms after a systemic febrile illness.

## Introduction

Meralgia paresthetica (MP) is a sensory mononeuropathy caused by entrapment or dysfunction of the lateral femoral cutaneous nerve (LFCN), a purely sensory nerve originating from the L2-L3 nerve roots [1,2]. The LFCN is particularly vulnerable to compression, as it traverses beneath or through the inguinal ligament near the anterior superior iliac spine. Clinically, MP presents with burning pain, numbness, paresthesia, or dysesthesia localized to the anterolateral thigh without motor weakness [1]. Common etiologies include obesity, diabetes mellitus, pregnancy, trauma, tight clothing, and postsurgical compression [2,3]. Less commonly, inflammatory or possible post-infectious neuropathic mechanisms have been proposed.

Piriformis syndrome (PS) is an uncommon neuromuscular condition caused by irritation or compression of the sciatic nerve by the piriformis muscle within the deep gluteal region [4,5]. Patients typically present with deep gluteal pain radiating to the posterior thigh, often exacerbated by prolonged sitting, hip movement, or ambulation. Because symptoms may mimic lumbar radiculopathy, diagnosis can be challenging [5].

Electrodiagnostic testing may support the diagnosis of MP, particularly in atypical or bilateral presentations. Seror and Seror demonstrated that abnormalities in sensory nerve conduction parameters and side-to-side asymmetry of the LFCN may support the diagnosis of MP [6]. Magnetic resonance imaging (MRI) may assist in excluding alternative diagnoses and identifying structural abnormalities associated with PS, including piriformis muscle edema, hypertrophy, or sciatic nerve impingement [7,8].

The coexistence of bilateral MP and unilateral PF is particularly unusual because these conditions involve anatomically distinct peripheral nerve entrapment mechanisms and may produce overlapping neuropathic and radicular-like symptoms, thereby creating diagnostic uncertainty. Furthermore, most reported cases of MP are mechanically mediated, whereas possible inflammatory or post-infectious associations remain less commonly recognized. Possible mechanisms linking a preceding febrile illness to focal neuropathies include immune-mediated inflammatory responses, perineural edema, and transient nerve susceptibility due to systemic cytokine activation, which may predispose peripheral nerves to entrapment at anatomically vulnerable sites. We report a case of bilateral MP with concurrent right-sided PS temporally associated with a preceding febrile illness, with the diagnosis supported by nerve conduction studies and pelvic MRI findings.

## Case presentation

A 30-year-old, previously healthy man presented with a 5-day history of fever, sore throat, generalized myalgia, and arthralgia. The febrile illness resolved spontaneously without specific treatment. Approximately one week later, he developed persistent aching discomfort and heaviness in both the lower limbs associated with intermittent muscle spasms and subjective low-grade evening fever. He also reported transient loose stools with mucus lasting three days.

Ten days after resolution of febrile illness, the patient developed burning pain and tingling over the anterolateral aspect of both thighs, which was more pronounced on the right side. He also complained of right gluteal pain aggravated by prolonged sitting and walking. There was no limb weakness, gait disturbance, bowel or bladder dysfunction, or lower back pain. The patient had no history of obesity, recent weight gain or loss, prolonged immobilization, occupational prolonged sitting, repetitive hip flexion activities, trauma, or use of tight garments or belts.

Neurological examination demonstrated preserved muscle bulk, tone, power, and deep tendon reflexes in both lower limbs. Sensory examination revealed allodynia and dysesthesia over the anterolateral aspect of both thighs in the distribution of the LFCN, with mild impairment of pinprick and light touch sensation in the same region, while vibration and proprioception were intact. Gait assessment revealed a normal, stable gait without an antalgic pattern or motor weakness. Examination of the gluteal region revealed no focal tenderness over the right piriformis region. However, the flexion-adduction-internal rotation (FAIR) test was positive on the right side, reproducing gluteal pain suggestive of PS. The straight-leg-raising test was negative bilaterally.

Routine laboratory investigations were largely unremarkable, except for a mildly elevated vitamin B12 level, which was not considered clinically explanatory for the presenting neurological condition (Table 1). Extended serological and autoimmune investigations, including dengue serology, coronavirus disease 2019 testing, human immunodeficiency virus serology, hepatitis screening, antinuclear antibody, and antineutrophil cytoplasmic antibody, were negative or within normal limits, making infectious, autoimmune, and vasculitic etiologies less likely (Table 2). Cerebrospinal fluid analysis demonstrated normal opening pressure, normal protein and glucose concentrations, absence of pleocytosis, and negative microbiological studies, with no evidence of infectious or inflammatory neuropathy (Table 3).

**Table 1:** Laboratory investigations with reference ranges

Investigation	Result	Reference Range
Hemoglobin	13.8	13–17 g/dL
Total leukocyte count	7	4–11 ×10⁹/L
Platelet count	245	150–450 ×10⁹/L
Fasting blood glucose	98	70–126 mg/dL
Glycated hemoglobin (HbA1c)	4.9	<6.5%
Serum creatinine	1.1	0.7–1.3 mg/dL
Blood urea nitrogen	14	7–20 mg/dL
Aspartate aminotransferase (AST)	28	<40 U/L
Alanine aminotransferase (ALT)	36	<40 U/L
Vitamin B12	956	200–900 pg/mL
Erythrocyte sedimentation rate (ESR)	8	<20 mm/hr
C-reactive protein (CRP)	2	<5 mg/L

**Table 2:** Serological and autoimmune investigations with reference ranges

Investigations	Result	Reference Range
Dengue NS1 antigen	Negative	Negative
Dengue IgM antibody	Negative	Negative
Dengue IgG antibody	Negative	Negative
SARS-CoV-2 antigen/PCR	Negative	Negative
SARS-CoV-2 IgM/IgG antibody	Negative	Negative
Human immunodeficiency virus (HIV) 1 and 2 serology	Negative	Negative
Hepatitis B surface antigen (HBsAg)	Negative	Negative
Hepatitis C antibody	Negative	Negative
Antinuclear antibody (ANA)	Negative	Negative
Antineutrophil cytoplasmic antibody (ANCA)	Negative	Negative
Rheumatoid factor	Negative	Negative
Serum complement C3	144	90–180 mg/dL
Serum complement C4	38	10–40 mg/dL
Serum folate	15.2	4-20 ng/mL
Serum protein electrophoresis	Normal	Normal pattern without monoclonal band

**Table 3:** Cerebrospinal fluid analysis

Parameter	Result	Reference Range
Opening pressure	10	6–20 cm H₂O
Appearance	Clear	Clear
White blood cells	0 cells/µL	0–5 cells/µL
Protein	23	15–45 mg/dL
Glucose	76	45–80 mg/dL
Gram stain/culture	Negative	Negative

Magnetic resonance imaging of the lumbosacral spine was unremarkable. Pelvic MRI demonstrated asymmetric enlargement and T2-weighted hyperintensity of the right piriformis muscle with adjacent soft tissue edema, findings compatible with PS (Figure 1).

**Figure 1:**
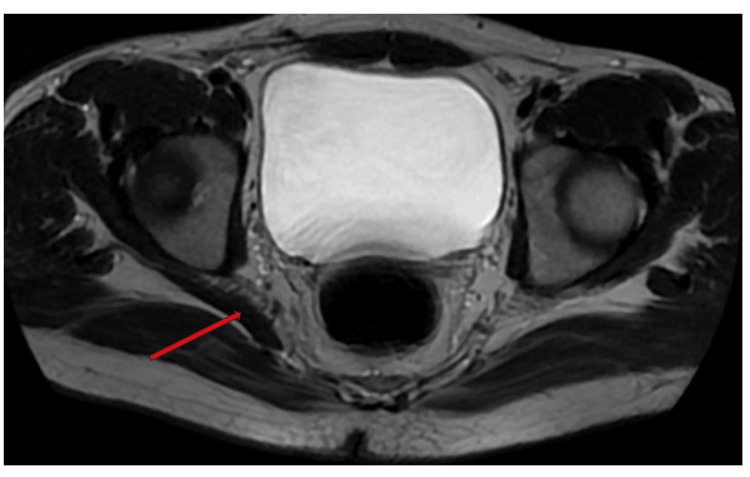
Axial T2-weighted pelvic MRI showing asymmetric enlargement and a hyperintense T2 signal of the right piriformis muscle (arrow) with surrounding deep gluteal soft tissue edema, findings compatible with right piriformis syndrome.

Nerve conduction studies demonstrated bilateral LFCN abnormalities consistent with MP, while pelvic MRI revealed findings compatible with right PS (Table 4). Motor nerve conduction studies were normal.

**Table 4:** Nerve conduction study findings demonstrating bilateral lateral femoral cutaneous nerve abnormalities consistent with meralgia paresthetica Institutional reference values: peak latency <5.0 ms, SNAP amplitude >5 µV, and conduction velocity >25 m/s. Abnormal lateral femoral cutaneous nerve conduction findings were characterized primarily by prolonged peak latency and reduced conduction velocity relative to institutional reference standards. All studies were performed with limb temperatures maintained at 32-34 °C.

Nerve	Side	Stimulation Site	Recording Site	Peak Latency (ms)	SNAP Amplitude (µV)	Distance	Conduction Velocity (m/s)
Sural	Right	Mid-Calf	Lateral Malleolus	3.5	13.1	14 cm	40
Sural	Left	Mid-Calf	Lateral Malleolus	3.5	13.8	14 cm	40
Lateral Femoral Cutaneous	Right	Inguinal Ligament Region	Anterolateral Thigh	5.7	7.9	12 cm	21.1
Lateral Femoral Cutaneous	Left	Inguinal Ligament Region	Anterolateral Thigh	5.9	7.8	12 cm	20.3

Because the abnormalities were bilateral, side-to-side comparison was of limited diagnostic value, and interpretation was based primarily on deviation from established laboratory reference standards. Electromyography was not performed; however, the absence of motor deficits, normal motor nerve conduction studies, and an unremarkable lumbosacral spine MRI made proximal lumbosacral radiculopathy unlikely.

The patient was treated with pregabalin 75 mg twice daily, nonsteroidal anti-inflammatory drugs, and physiotherapy focusing on piriformis stretching and core-strengthening exercises. His symptoms improved significantly over two weeks, with complete resolution of burning pain in the thigh and marked improvement in gluteal discomfort at follow-up. A clinical timeline summarizing symptom progression, diagnostic evaluation, and treatment response is presented in Figure 2.

**Figure 2:**
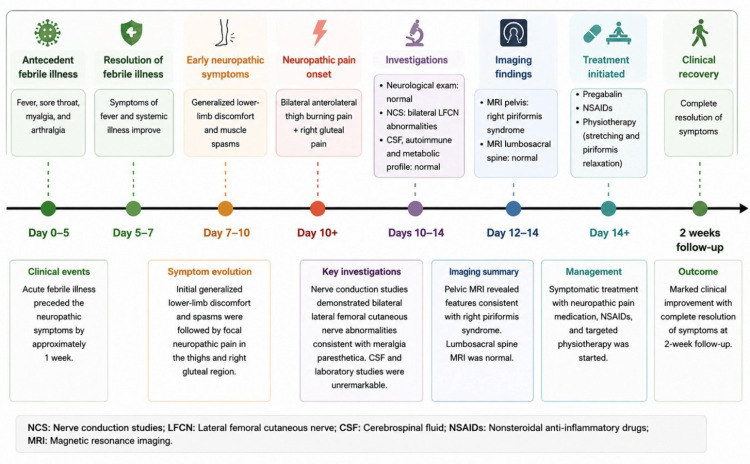
Clinical timeline of symptom onset, investigations, imaging, treatment, and recovery

## Discussion

This case demonstrates the unusual coexistence of bilateral MP and right PS temporally associated with a preceding febrile illness in a previously healthy individual. Although MP and PS are individually recognized peripheral nerve entrapment syndromes, their concurrent presentation has rarely been described in the literature and may pose substantial diagnostic challenges because of overlapping sensory and radicular-like symptoms.

MP is a mononeuropathy involving the LFCN, classically attributed to focal mechanical compression at the inguinal ligament. Established risk factors include obesity, diabetes mellitus, pregnancy, trauma, prolonged external compression, and tight garments [1,2]. However, emerging evidence suggests that inflammatory, immune-mediated, and non-mechanical etiologies may also contribute to LFCN dysfunction [3,9,10]. Recent reviews suggest that infectious or post-infectious inflammatory processes may contribute to focal mononeuropathies through immune-mediated nerve injury, perineural edema, or transient inflammatory entrapment phenomena [3,9]. In the present case, the close temporal relationship between the antecedent febrile illness and subsequent onset of neuropathic symptoms raises the possibility of a post-infectious inflammatory mechanism affecting multiple peripheral nerve territories.

The diagnosis of MP remains primarily clinical and is characterized by burning pain, paresthesia, numbness, or dysesthesia involving the anterolateral thigh without motor weakness. Electrodiagnostic studies may provide supportive evidence in atypical or bilateral cases [6,11]. In our patient, bilateral abnormalities on sensory nerve conduction studies confirmed LFCN involvement. Seror and Seror demonstrated that abnormalities in LFCN sensory conduction parameters, including prolonged latency, reduced conduction velocity, and altered sensory nerve action potential amplitudes, may support the diagnosis of MP, particularly in diagnostically challenging presentations [6]. Furthermore, contemporary reviews emphasize that electrodiagnostic testing is particularly useful when bilateral disease, proximal pathology, or overlapping lumbar disorders are suspected [9,11].

PS is a controversial and frequently underrecognized cause of deep gluteal pain and extraspinal sciatica resulting from irritation or compression of the sciatic nerve by the piriformis muscle [4,5]. Diagnostic difficulty arises because PS often mimics lumbosacral radiculopathy, sacroiliac dysfunction, or other deep gluteal syndromes [4,5,12]. In our patient, MRI demonstrated asymmetric edema and enlargement of the right piriformis muscle, supporting the diagnosis of PS. Previous imaging studies have shown that MRI and MR neurography may identify piriformis hypertrophy, muscle edema, sciatic nerve hyperintensity, and morphometric abnormalities associated with sciatic nerve irritation [7,8,12,13]. Recent narrative reviews further support the growing role of advanced imaging in improving diagnostic confidence in PS, particularly when physical examination findings are inconclusive [12].

Several important differential diagnoses were carefully excluded during evaluation. Normal motor strength, preserved deep tendon reflexes, normal cerebrospinal fluid findings, and an unremarkable lumbosacral spine MRI argued against lumbosacral radiculopathy, inflammatory demyelinating polyradiculoneuropathy, plexopathy, or generalized peripheral neuropathy. In addition, the absence of diabetes mellitus, obesity, recent trauma, surgery, or external compressive factors reduced the likelihood of conventional mechanical causes of MP. The coexistence of bilateral sensory mononeuropathy and ipsilateral deep gluteal neuropathic pain further supported the possibility of a multifocal inflammatory neuropathic process rather than isolated mechanical entrapment alone.

The potential association between antecedent infection and subsequent peripheral neuropathic syndromes has been increasingly recognized. Post-infectious immune-mediated neuropathies may occur through molecular mimicry, inflammatory cytokine activation, or transient autoimmune responses directed against peripheral nerves [3,9]. Although cerebrospinal fluid analysis and autoimmune screening were unremarkable in this case, the absence of identifiable metabolic, compressive, structural, or systemic inflammatory etiologies makes a post-infectious inflammatory process a plausible consideration. Although a definitive causal relationship cannot be established, the temporal association between the antecedent febrile illness and the subsequent onset of neuropathic symptoms may suggest a post-infectious inflammatory process; however, this remains speculative in the absence of identifiable infectious or immunological markers.

The management of both MP and PS is generally conservative and includes neuropathic pain control, physiotherapy, stretching exercises, activity modification, and avoidance of aggravating factors [1,4,9,12]. Pregabalin was selected for our patient because of its efficacy in neuropathic pain modulation, and targeted physiotherapy was initiated to address piriformis-related sciatic irritation and functional impairment. The patient demonstrated marked clinical improvement with multimodal conservative treatment, emphasizing the importance of early recognition and appropriate non-surgical management.

The clinical significance of this case lies in the coexistence of two anatomically distinct peripheral nerve entrapment syndromes occurring shortly after a self-limited febrile illness in the absence of conventional mechanical risk factors. Previously reported post-infectious neuropathic presentations have more commonly involved generalized peripheral neuropathies, mononeuritis multiplex, cranial neuropathies, or inflammatory polyradiculopathies rather than simultaneous focal entrapment neuropathies affecting separate peripheral nerve territories. The combination of bilateral LFCN involvement with unilateral piriformis-related sciatic irritation, therefore, broadens the spectrum of neuropathic presentations that may occur in temporal association with systemic inflammatory or infectious illnesses.

To our knowledge, reports describing simultaneous bilateral MP and unilateral PS temporally associated with a preceding febrile illness remain extremely limited in the literature. This case expands the spectrum of possible inflammatory or post-infectious neuropathic associations and highlights the importance of considering concomitant entrapment neuropathies in patients presenting with atypical lower-limb sensory symptoms following systemic febrile illness. The limitations of this report include the inability to establish definitive causality between the antecedent febrile illness and the subsequent neuropathic syndromes, as well as the absence of serological confirmation of a specific infectious trigger.

## Conclusions

Bilateral meralgia paresthetica with concurrent piriformis syndrome represents an uncommon clinical presentation temporally associated with a preceding self-limited febrile illness. Overlapping lower-limb sensory and gluteal symptoms may mimic lumbosacral radiculopathy and other neuropathic disorders, creating diagnostic challenges. Careful neurological examination supported by electrodiagnostic studies and pelvic MRI can facilitate accurate diagnosis and guide effective conservative management. This case highlights the importance of considering coexisting peripheral nerve entrapment syndromes and possible post-infectious inflammatory mechanisms in patients presenting with atypical lower-limb neuropathic symptoms.
